# Imaging the living plant cell: From probes to quantification

**DOI:** 10.1093/plcell/koab237

**Published:** 2021-09-29

**Authors:** Leia Colin, Raquel Martin-Arevalillo, Simone Bovio, Amélie Bauer, Teva Vernoux, Marie-Cecile Caillaud, Benoit Landrein, Yvon Jaillais

**Affiliations:** Laboratoire Reproduction et Développement des Plantes, Université de Lyon, ENS de Lyon, CNRS, INRAE, 69342 Lyon, France; Laboratoire Reproduction et Développement des Plantes, Université de Lyon, ENS de Lyon, CNRS, INRAE, 69342 Lyon, France; Laboratoire Reproduction et Développement des Plantes, Université de Lyon, ENS de Lyon, CNRS, INRAE, 69342 Lyon, France; LYMIC-PLATIM imaging and microscopy core facility, Univ Lyon, SFR Biosciences, ENS de Lyon, Inserm US8, CNRS UMS3444, UCBL-50 Avenue Tony Garnier, 69007 Lyon, France; Laboratoire Reproduction et Développement des Plantes, Université de Lyon, ENS de Lyon, CNRS, INRAE, 69342 Lyon, France; Laboratoire Reproduction et Développement des Plantes, Université de Lyon, ENS de Lyon, CNRS, INRAE, 69342 Lyon, France; Laboratoire Reproduction et Développement des Plantes, Université de Lyon, ENS de Lyon, CNRS, INRAE, 69342 Lyon, France; Laboratoire Reproduction et Développement des Plantes, Université de Lyon, ENS de Lyon, CNRS, INRAE, 69342 Lyon, France; Laboratoire Reproduction et Développement des Plantes, Université de Lyon, ENS de Lyon, CNRS, INRAE, 69342 Lyon, France

## Abstract

At the center of cell biology is our ability to image the cell and its various components, either in isolation or within an organism. Given its importance, biological imaging has emerged as a field of its own, which is inherently highly interdisciplinary. Indeed, biologists rely on physicists and engineers to build new microscopes and imaging techniques, chemists to develop better imaging probes, and mathematicians and computer scientists for image analysis and quantification. Live imaging collectively involves all the techniques aimed at imaging live samples. It is a rapidly evolving field, with countless new techniques, probes, and dyes being continuously developed. Some of these new methods or reagents are readily amenable to image plant samples, while others are not and require specific modifications for the plant field. Here, we review some recent advances in live imaging of plant cells. In particular, we discuss the solutions that plant biologists use to live image membrane-bound organelles, cytoskeleton components, hormones, and the mechanical properties of cells or tissues. We not only consider the imaging techniques per se, but also how the construction of new fluorescent probes and analysis pipelines are driving the field of plant cell biology.

## Teaser

Specific examples are used to illustrate some of the challenges of live cell imaging, from designing genetically encoded probes to choosing a pipeline for image analysis and quantification.

## Introduction

As recently described by Marc Somssich in his “short history of plant light microscopy”, the invention of the microscope and its use to observe plant tissues “opened up a completely new world previously hidden to the human eye” (Somssich, 2021). It notably led to the cell theory, which proposed that the cell is the fundamental unit of life and placed the cell at the center of organismal biology (Kierzkowski and Routier-Kierzkowska, 2019). In this review, we focus on the recent advances made in the field of live imaging of plant cells.

From the point of view of probes, live imaging of plants, as in the rest of biology, was really boosted by the discovery and use of fluorescent proteins (Chalfie, 2009; Somssich, 2021). While new, improved fluorescent proteins in different colors are continuously being developed (Lambert, 2019), most of the recent advances came from the development of genetically encoded biosensors and reporters (Grossmann et al., 2018; Walia et al., 2018); we will describe some of these advances here. On the microscopy side, confocal microscopy is the most widely used method by far. Briefly, this technique relies on one or several pinholes that block out-of-focus light and thus increase the contrast and resolution of fluorescent imaging by collecting only (or mostly) the light coming out of the focal plane (Table 1; Bayguinov et al., 2018). Confocal microscopy is particularly well suited for imaging moderately thick and rather transparent samples, such as a variety of plant tissues or organs. We will also introduce some of the new imaging techniques that have increased the speed of acquisition, its sensitivity, spatial resolution, or depth of acquisition (Table 1; Grossmann et al., 2018; Clark et al., 2020).

**Table 1:** Light microscopy techniques described in this review

Microscopy technique	Principle	Advantages and limitations
Confocal Laser Scanning Microscopy (CLSM)	The sample is scanned point-by-point by a focused laser beam (raster scanning), out-of-focus signal is removed by an adjustable iris (i.e. pinhole), and an image is built up pixel-by-pixel by collecting the emitted light via sensitive point detectors (e.g. PMTs)	Versatile technique, as it works with both thick and thin samples and with many different objective magnifications (i.e. variable pinholes), can produce thin optical sections, can spectrally separate different fluorophores, and the focused laser beam is compatible with photoactivation or targeted photobleaching. However, the application of an intense and focused laser beam can lead to photodamage and photobleaching, and scanning the entire sample in 3D is relatively slow
Spinning Disk Confocal Microscopy (SDCM)	Excitation light passes through a series of pinholes on a rotating disk so that only the imaged pixels are illuminated at a given time, out-of-focus light is also removed by those pinholes and light is collected in parallel on sensitive array detector(s) (e.g. EMCCD or sCMOS camera)	Faster and more gentle imaging than CLSM at the expense of *z*-resolution (i.e. optical section not as thin as with CLSM), difficult to perform spectral imaging, need additional dedicated equipment for photomanipulation. Not as versatile as CLSM because it has fixed pinholes that are not adjustable to various objective magnifications
TIRFM	The laser beam hits the coverslip/medium interface at a critical angle, leading to its total refraction, which locally emits a shallow evanescent wave (∼100–200 nm). As a result, only the portion of the cell in close contact with the coverslip is illuminated	Because there is no out-of-focus light, TIRF microscopes can be coupled with highly sensitive cameras, thereby allowing very fast acquisition as well as single molecule imaging. TIRFM increases the resolution in depth (basically determined by the thickness of the evanescent wave); however, this technique is limited to the cell cortex
Variable Angle Epifluorescence Microscopy (VAEM/VA-TIRF)	Variation of the TIRF technique that uses a subcritical angle for the laser beam, which does not lead to total refraction, but instead partial (inclined) illumination of the sample	VA-TIRF/VAEM is sometimes referred to as the “dirty” TIRF technique. It is a compromise between a deeper excitation into the sample and a less contrasted image
Light Sheet Fluorescence Microscopy (LSFM)	The whole field-of-view is illuminated by a laser light sheet (i.e. thin slice of light of a few hundreds of nanometers to few micrometers) perpendicularly to the direction of the detection	LSFM is very rapid and gentle in terms of phototoxicity and photobleaching, thus it allows long term imaging, or fast 4D imaging. Like SDCM, LSFM cannot perform spectral imaging easily and needs additional dedicated equipment for photomanipulation. Sample mounting can be difficult and often highly specialized, which means that LSF microscopes are often dedicated to specific applications and not highly versatile
Two-photon Excitation Microscopy (TPEM)	Simultaneous excitation of a fluorophore by two photons with longer wavelength than the emitted light. It typically uses tunable femtosecond pulsed laser with a raster scanning as in CLSM	Two-photon microscopy is used for deep tissue imaging, as near infrared light minimize scattering in the tissue and only the fluorophores in the focal plan are activated. High laser energy can destroy the cell by overheating, which is a potential drawback, but it can be used to generate very precise cell ablation deep in the tissue. Many dyes are excited by the same wavelength in TPEM, which can generate strong background and reduces the choice of fluorophores for multicolor imaging
PALM	Super-resolution microscopy technique based on stochastic activation of photo-activatable fluorescent proteins, which allows their precise localization. Images are reconstituted by iterative cycles of activation, acquisition, and photobleaching	PALM has a very high lateral resolution (∼20–30 nm) and is a single molecule imaging technique (as such, it is often performed in TIRF, which is a very sensitive imaging technique). However, it is very slow because it requires iterative image acquisition, and the cells receive a lot of laser power (photodamage). It also requires dedicated transgenic lines expressing photo-activatable or photo-switchable fluorescent protein fusions, and multicolor imaging is limited. PALM also requires a lot of post-acquisition processing
Structured Illumination Microscopy (SIM)	Super-resolution imaging technique that uses interference patterns created by a grid. It requires several images (with translations and rotations of the grid) and post-processing to compute a super-resolved image	SIM roughly double the resolution limit of light microscopy (∼120 nm laterally, 300 nm axially). It can be done in 3D and with multiple fluorophores and is compatible with classical fluorescent proteins. Because several images need to be acquired, it can be slow, it requires image post-processing and somewhat long illumination time (photobleaching). The increase in resolution is not as high as in PALM. Note that it can be coupled with TIRF (TIRF-SIM) to increase the contrast
SCLIM	Spinning disk microscopy with several paralleled array detectors and post processing (i.e. deconvolution)	SCLIM is equipped with three array detectors (i.e. cameras), and as such it is fast and can acquire several channels simultaneously, making it a solution of choice to study rapid processes such as membrane trafficking. However, it relies heavily on image post-processing, and the increase in lateral resolution is due to the deconvolution algorithm and is thus modest
Stimulated Emission Depletion (STED) microscopy	Scanning of the sample by two different laser pulses: a first excitation pulse (excitation laser), and a second doughnut-shaped pulse (depletion laser) for the selective deactivation of the fluorophore. The focal spot is raster scanned, like in CLSM	Lateral resolution of ∼50–70 nm (>500 nm axially), can be rapid but in a small field-of-view, deep imaging compared with other super-resolution techniques (10- to 15-µm deep) and does not require image post-processing. Has not been extensively used in live imaging in plants, likely due to high phototoxicity (high-intensity depletion laser) and photobleaching
Fluorescence Recovery After Photobleaching (FRAP)	Technique used to study fluorescent molecule diffusion based on the bleaching of a population of fluorophores and the subsequent quantitative analysis of the fluorescence recovery.	FRAP is a popular technique to study molecule diffusion because it can be performed on most CLSM and with standard fluorescent protein fusions. It provides information on the diffusion of an ensemble of molecules, but diffusion coefficient calculation requires complicated models (and thus is quite indirect).
Single Particle Tracking (SPT)	Technique aiming at tracking single fluorescent particles (e.g. single molecules or single objects such as vesicles or microtubule tips) to analyze their dynamics. Can be coupled with PALM (i.e. sptPALM) to obtain super-resolved localization of diffusing individual molecules	SPT techniques are a direct measure of diffusion and they tend to be very accurate for relatively slow diffusing molecules/structures compared to other techniques. They rely on complex image post-processing: automated tracking algorithms. These algorithms work well only if individual structures are well-defined/isolated from each other
FRET	Energy transfer between a donor and acceptor fluorescent protein that happens when they are in close proximity (i.e. less than 10 nm) and at the correct orientation with respect to each other	FRET is typically used as a ruler to study molecular proximity, for example to study protein-protein interactions, or intramolecular conformational changes in the case of ratiometric biosensors. It is a very powerful technique, as it can detect dynamic molecular interactions in vivo. FRET can be measured on a variety of microscopes (including CLSM and widefield microscopy). However, it is difficult to accurately measure in practice. In addition, it is difficult to predict a priori how well FRET will work between two interacting molecules, and it has to be tested empirically
FLIM	Technique based on the exponential decay rate of a fluorophore, which requires the use of a pulsed illumination source	FLIM is often used to accurately measure FRET, since the fluorescent lifetime of the donor decreases upon energy transfer. It can also be used to differentiate fluorophores with otherwise overlapping spectra and can (for example) help to filter out autofluorescence. Although they are becoming more and more accessible, most FLIM systems are complex to use both in terms of image acquisition and analyses

There are already a number of excellent reviews that discuss live imaging in plants (see, e.g. Sappl and Heisler, 2013; Berthet and Maizel, 2016; Grossmann et al., 2018; Komis et al., 2018; Clark et al., 2020). Here, rather than having a mostly technical and technological focus, we decided to consider some of the classical problems in cell biology to illustrate (1) how plant biologists use live imaging to address them, (2) what are the challenges in setting up live imaging experiments, and (3) what are the solutions to overcome these pitfalls. To this end, we will review some of the methods used to image the cytoskeleton, the plant endomembrane network, and plant hormones and their activity. Finally, we will introduce an array of imaging techniques that are being developed to study the biophysical and mechanical properties of plant cells and tissues.

## Visualization and quantification of the plant cytoskeleton

### Markers for live imaging of the cytoskeleton

Actin and microtubule filaments are among the most fascinating structures in the cell. They are highly dynamic and under constant remodeling, which quickly prompted the development of live reporters to capture these ever-changing structures. In plants, one of the more reliable actin reporters and one of the first to be described is the *Arabidopsis thaliana* Fimbrin-like, AtFim1 (Table 2; McCurdy and Kim, 1998; Kovar et al., 2001; Voigt et al., 2005). The C-terminal half of AtFim1 (aa 325–687; coined AtFim1 ACTIN-BINDING DOMAIN2 [fABD2]) fused to a fluorescent protein is more efficient at labeling the actin filaments than the full-length protein and is therefore generally used as a standard for actin filament visualization in vivo (Ketelaar et al., 2004; Sheahan et al., 2004; Wang et al., 2004). While the use of the mouse Talin as a reporter has rapidly diminished due to side effects, the fABD2 domain has been largely used to visualize the actin cytoskeleton (Wang et al., 2004). Yet, the strong expression of the *GFP‐fABD2‐GFP* reporter has inhibitory effects on cell and organ growth; therefore, it is crucial to use promoters with low or moderate expression levels (Wang et al., 2008; Dyachok et al., 2014). The other commonly used reporter for actin filaments is a 17-amino acid (aa) peptide named LifeAct, which appears to be a faithful biosensor without extensively disrupting the dynamics of the actin filaments (Riedl et al., 2008). While LifeAct decorates actin filaments with minimum perturbation of their dynamics, *LifeAct* expression also needs to be optimized to reach an expression level lower than for *fADB2* to prevent the bundling of actin filaments (Era et al., 2009; Dyachok et al., 2014). The dynamic reorganization of the actin cytoskeleton can be assessed at super-resolution by photoactivation localization microscopy, with the LifeAct reporter fused to a photoactivatable fluorescent protein (Durst et al., 2014).

**Table 2:** Commonly used cytoskeleton markers in *A. thaliana*

Cytoskeleton	Sensor name	Sensor type	Construct	Comments	Ref. of transgenic line	NASC Stock #
Actin	AtFim1	Actin binding	Full-length AtFim1	Induce morphological defect at high expression	Wang et al., 2004; Sheahan et al., 2004	–
	fABD2	Actin binding	C-terminal half of AtFim1 (aa 325–687)	Induce morphological defect at high expression	Wang et al., 2004; Sheahan et al., 2004; Ketelaar et al., 2004	N799991
	LifeAct	Actin binding	Actin-binding peptide (17 aa) of yeast abp140p	Induce morphological defect at high expression	Era et al., 2009	–
Microtubule	MBD	Microtubule binding	Human MAP4	Enhanced microtubule polymerization, nucleation, bundling, and stabilization	Marc et al., 1998	N799990
	MAP65-1	Microtubule binding	MAP of 65 kDa-1	Enhanced microtubule polymerization, nucleation, bundling, and stabilization	Lucas et al., 2011	N67830
	TUA6	Direct microtubule labeling	TUBULIN alpha 6 gene	Microtubule and cytoplasmic localization	Ueda et al., 1999	N6551
	TUA5	Direct microtubule labeling	TUBULIN alpha 5 gene	Microtubule and cytoplasmic localization	Liu et al., 2016	–
	TUB6	Direct microtubule labeling	TUBULIN beta 6 gene	Microtubule and cytoplasmic localization	Nakamura et al., 2004	N6550; N67065; N67065
	EB1	Plus-end microtubule tip	Arabidopsis End-Binding Protein-1a	Plus end tip of the growing microtubules	Chan et al., 2003	–

Table listing some of the commonly used genetically encoded cytoskeleton markers. Fim, FIMBRIN-LIKE; ABD, actin binding domain; EB1, END-BINDING1.

Like for the actin cytoskeleton, visualization of microtubules in vivo is often based on a fluorophore-conjugated microtubule-associated protein (MAP). As such, the microtubule-binding domain (MBD) of the human MAP-4 fused to GFP became a typical reporter used to visualize microtubules in vivo (Table 2; Marc et al., 1998). Other constructs with plant MAPs are also available, such as the MAP of 65 kDa-1, MAP65-2, or MAP65-4 (Fache et al., 2010; Lucas et al., 2011; Creff et al., 2015; Boruc et al., 2017). In these cases, careful attention needs to be taken in the interpretation of the results, since such proteins enhance microtubule polymerization and promote their nucleation, bundling, and stabilization (Fache et al., 2010). The level of expression of such reporters should therefore be tightly monitored, as developmental defects such as dwarfism or organ twisting are observed when their expression is too high (Holzinger et al., 2009). Another approach is to directly tag the tubulin monomer itself (Ueda et al., 1999). Fusions of Tubulin Alpha 6 (TUA6), TUA5, and Beta 6 (TUB6) subunits to various fluorescent tags are used to describe the organization and dynamics of microtubules in planta (Ueda et al., 1999; Nakamura et al., 2004; Abe and Hashimoto, 2005; Liu et al., 2016). However, depending on the experiments and expression levels, the fluorescent signal may appear more cytoplasmic using TUA6/TUB6 than MBD-based reporters (Doumane et al., 2021). This makes quantification trickier, especially for automatic detection of individual microtubule bundles, but at the same time, TUA6/TUB6 markers induce fewer side-effects and developmental phenotypes than MBD-based reporters. Nonetheless, as discussed for previous reporters, high expression of TUA6 or TUB6 may still induce phenotypes, for example on cell wall synthesis (Abe and Hashimoto, 2005; Burk et al., 2006).

While the markers described above are used to visualize the entire microtubule, some reporters target subdomains of the microtubule, such as Arabidopsis End-Binding Protein-1a (35Spro:AtEB1a-GFP, Chan et al., 2003). This protein labels the plus-ends of microtubules and is visualized as a comet-like structure corresponding to the tip of the growing microtubule (Chan et al., 2003; Bisgrove et al., 2008; Galva et al., 2014; Wong and Hashimoto, 2017; Elliott and Shaw, 2018; Molines et al., 2018, 2020). This tool is particularly useful to address the rate of microtubule growth or the angle between branched microtubules in a given tissue or condition (Chan et al., 2009; Montesinos et al., 2020).

Whenever possible, it is best to use multiple markers to interpret live imaging experiments based on both actin and microtubule fluorescent reporters. It is also important to keep in mind that cytoskeleton reporters might not label the entire population of microtubules or actin filaments due to competition with endogenous cytoskeleton regulators (Sadot and Blancaflor, 2019). As such, accurate detection of the cytoskeleton network by immunolocalization should also be considered as an alternative (Belcram et al., 2016; Tichá et al., 2020; Du et al., 2021), although it is not compatible with live imaging. In the animal field, vital fluorescent dyes that can be added to the culture medium that label either actin or microtubules, such as SiR-actin or Sir-tubulin, are becoming popular due to their ease of use (i.e. no need to genetically express a reporter; Lukinavičius et al., 2014; Melak et al., 2017). To our knowledge, these dyes have not been extensively used in plant systems, very likely because they do not enter the cells, perhaps due to the presence of the cell wall. In any case, like for genetically encoded markers, these chemical probes also tend to affect cytoskeleton dynamics (Melak et al., 2017). Other technical challenges are still blocking progress in the field, in particular the loss of the fluorescent signal intensity in the inner tissues. The development of fluorescent markers expressed under the control of tissue-specific promoters might help in this matter, as was done in the study of lateral root initiation (Barro et al., 2019). Alternatively, the use of two-photon microscopy might help to penetrate deeper into thick tissues (Table 1; Grossmann et al., 2018; Mizuta, 2021).

### Model systems for live imaging of the cytoskeleton

The cytoskeleton is very important for cell differentiation, elongation, and polarity. While live imaging of cytoskeleton components has been carried out in many different cell types, it is worth mentioning the few model systems that have been recurrently used over the years by different groups. For example, root hairs and pollen tubes have extensively been used to study cytoskeleton dynamics in tip growing cells (Ketelaar, 2013; Scholz et al., 2020; Xu and Huang, 2020). The tobacco (*Nicotiana tabacum*) pollen tube is, in particular, an excellent model for live imaging studies of tip growth because they are big cells that are easy to transform and to image (Kost et al., 1998; Klahre and Kost, 2006; Scholz et al., 2020; Xu and Huang, 2020; Fratini et al., 2021). Microtubules are critical for anisotropic growth, which has been extensively studied in the hypocotyl (Shaw, 2013; Lenarcic et al., 2017). The cytoskeleton is also important for cell wall differentiation, which has been studied using a variety of systems, including transient expression in *Nicotiana* *benthamiana* leaves and in differentiating xylem (Oda and Fukuda, 2012; Oda, 2015). Of note, a cellular system was recently established to study long-term microtubule rearrangements occurring during proto-xylem development (Schneider et al., 2021). This system, based on xylem trans-differentiation upon induction of the transcription factor VASCULAR-RELATED NAC DOMAIN7, allows microtubule dynamics to be followed at high temporal resolution and over the course of several hours.

The cytoskeleton is also extremely dynamic and essential during cell division. Historically, live imaging of cell division has been performed using cell cultures such as tobacco BY-2 cells (Buschmann, 2016). The maize (*Zea mays*) leaf is another system used to study cytoskeleton dynamics during cell division (Rasmussen, 2016; Martinez et al., 2017). In Arabidopsis, the shoot apical meristem has been used to study the link between cell division orientation, microtubule dynamics, and mechanical forces (Louveaux and Hamant, 2013; Louveaux et al., 2016). The shoot apical meristem is indeed an excellent model system for many live imaging approaches, including cytoskeleton visualization and the study of cell division (Grandjean et al., 2004; Heisler and Ohno, 2014; Tobin and Meyerowitz, 2016; Willis et al., 2016; Hamant et al., 2019a). This is because (1) it develops relatively slowly and thus does not require fast imaging systems, (2) its morphogenesis is mainly driven by events happening in the epidermis (L1 layer) (Kutschera and Niklas, 2007; Savaldi-Goldstein et al., 2007; Vernoux et al., 2021), which is easily amenable to light microscopy approaches and can be targeted by drugs or exogenous hormonal treatments (Grandjean et al., 2004; Echevin et al., 2019; Brunoud et al., 2020), and (3) it can be excised from the plant and grown in vitro for a few days (Grandjean et al., 2004; Brunoud et al., 2020).

The root, particularly the root tip, is generally considered a model of choice by plant cell biologists. This is because the root tip is thin and transparent (without the autofluorescence of the chloroplasts), with cells that are not yet fully differentiated (and thus have small vacuoles, an expended cytoplasm, and a thin cell wall with reduced autofluorescence) and relatively slow cytoplasmic streaming. However, this model still has some limitations. First, the root tip very quickly grows out of the field of view (in roughly 30 min), which limits long time-lapse approaches, for example to study cell division. This problem can now be solved using unsupervised approaches to track root growth (Doumane et al., 2017; von Wangenheim et al., 2017). For example, using genetically encoded actin reporters and automatic root tracking, actin dynamics was recently imaged and quantified during plant cell division at unprecedented time scales (Lebecq et al., 2021). Second, roots constantly reorient their growth according to the gravity vector (Armengot et al., 2016), a response that is blocked when slides are mounted horizontally.

### Quantification of cytoskeleton dynamics in live imaging experiments

With recent advances in live-cell imaging, huge amounts of data are now generated for each experiment. Post-acquisition processing and quantitative analysis of the dynamics and organization of the cytoskeleton are the most time-consuming parts of the experimental procedure. Indeed, quantitative information is now becoming the standard to study the architecture and dynamics of the cytoskeleton (Autran et al., 2021). Quantification of cytoskeleton dynamics is generally obtained through the analysis of time sequences obtained either on single images or projected *z*-stacks. Using color-coded image sequence in the widely used image analysis software ImageJ (Schindelin et al., 2012; Schneider et al., 2012), the shift in the positions of bundles in interphasic cells can be visualized (Kuběnová et al., 2021). This post-acquisition analysis can be coupled with the generation of a kymograph, depicting straight lines when the cytoskeleton is immobile and wavy lines in the case of active movements (Lindeboom et al., 2013; Doumane et al., 2021; Schneider et al., 2021). The degree of bundling of the cytoskeleton in normalized image stacks can be obtained in a semi-automated way, using a plot profile generated from the Gel Analyser ImageJ function (Molines et al., 2018). This simple method allows one to rapidly compare the degree of bundling under different conditions or in genetic backgrounds expressing the same fluorescent reporter. Further parameters can be extracted from time series, such as the growth and shrinkage speed or the catastrophe and rescue rates (Lindeboom et al., 2013; Schneider et al., 2019). Importantly, at the subcellular level, in vivo imaging and quantification of the cytoskeleton in three dimensions is still challenging. Collaborative projects between cell biologists and mathematicians with expertise in image analysis might go a long way toward filling this gap.

**Figure 1:**
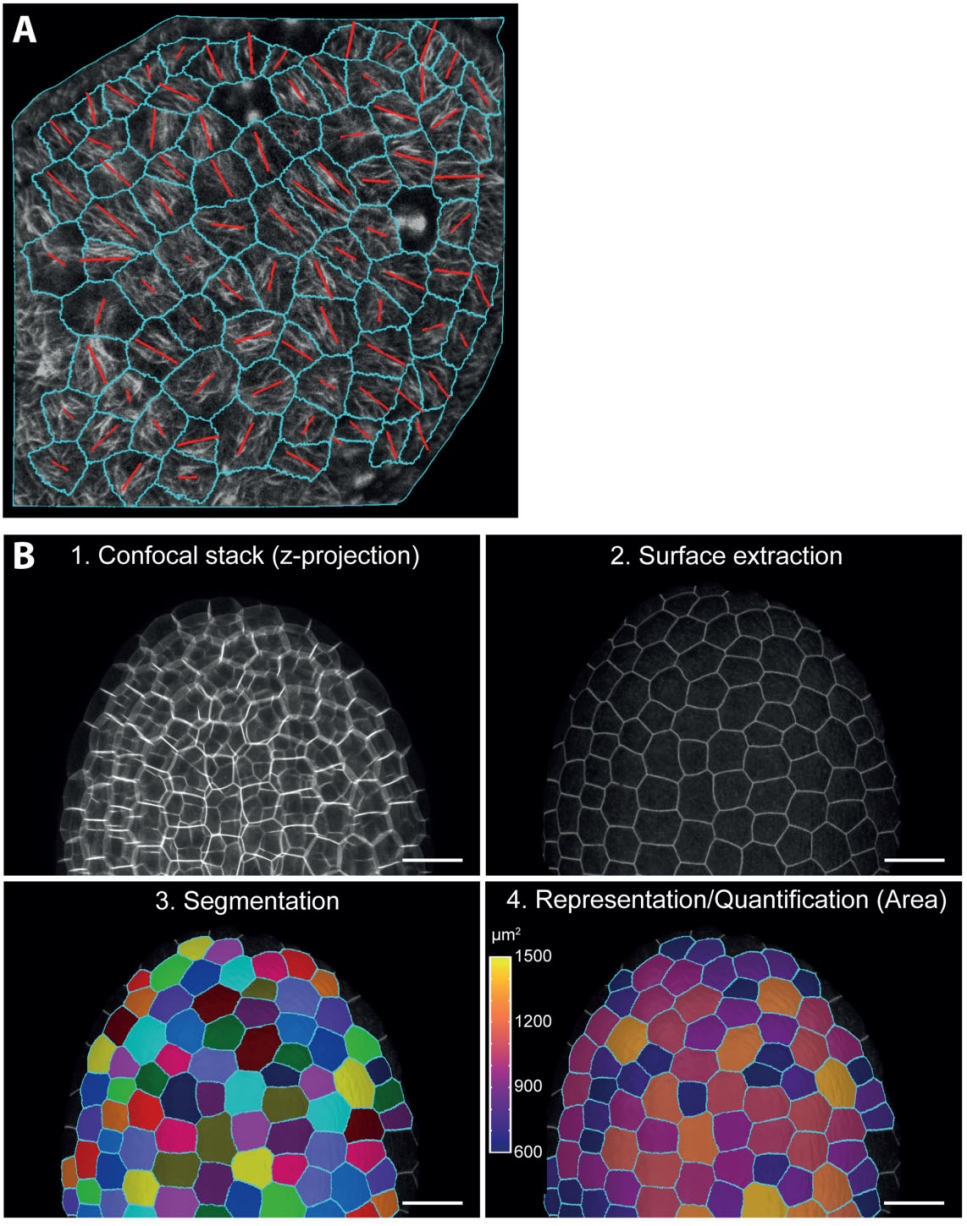
Examples of image analysis using the developing Arabidopsis seed as a model. A, Analysis of microtubule organization (MAP65-1-RFP) in a developing Arabidopsis seed at 2 days after pollination (DAP) with FibriTool and MorphographX. Scale bar, 10 µm. The orientation and length of the red bar represent the mean orientation and degree of organization of the microtubule array in a given cell, respectively. B, Segmentation of a confocal stack of a developing Arabidopsis seed (5 DAP) expressing LTi6b-GFP analyzed using the level set method (LSM) (Kiss et al., 2017) and MorphographX (de Reuille et al., 2015). Scale bar, 50 µm.

One of the standards for the quantitative measurement of cytoskeleton organization and thereby cell growth anisotropy is the ImageJ plugin FibrilTool (Boudaoud et al., 2014). This computing method assesses the pixel intensity level in a region of interest (ROI) and generates a vector tangent to the fibrils, giving us access to the anisotropy of the network in a semi-automatic manner (Boudaoud et al., 2014; see Figure 1A for an example). Such an approach has been successfully used to study the anisotropy of the microtubule network after genetic perturbation or pharmacological treatment in different systems (Robinson and Kuhlemeier, 2018; Riglet et al., 2020; Zhao et al., 2020). Similar approaches were recently used to quantify how geometry affects cytoskeletal organization by confining single cells (or protoplasts) within microfabricated microwells of various geometries (see Colin et al. (2020), Durand-Smet et al. (2020), and the last paragraph of this review). This plugin has been integrated into the MorphographX platform (de Reuille et al., 2015), thus allowing microtubule organization on computer-assisted cell segmentations to be analyzed (see next paragraph).

## Live imaging of membrane lipids and organelles

### Imaging the plasma membrane, a key to segmenting cells in tissues

The ability to segment cells is crucial for morphodynamic approaches, and having good markers specific to the cell contour is a pre-requisite for automatic segmentations (Hong et al., 2018). It is possible to segment cells by labeling the cell wall. In particular, propidium iodide (PI) is a red fluorescent dye that labels pectins in the cell wall and is often used in live imaging approaches to label cell contour (Kierzkowski et al., 2019; Sede et al., 2020); however, it is toxic to cells and affect growth, thus limiting long-term live cell imaging. Alternatively, membrane dyes or fluorescently tagged plasma membrane proteins are often used to segment cells when performing live imaging of growing tissues. A popular dye used to label the plasma membrane is FM4–64. This dye can be directly applied to live cells or tissues because it fluoresces only in a lipidic environment (Grandjean et al., 2004; Rigal et al., 2015; Doumane et al., 2017). An important property of FM4–64 is that it cannot pass through membrane. Thus, when applied to the imaging medium, it first labels the plasma membrane before labeling internal compartments following endocytosis. FM4–64 and PI are convenient because they fluoresce in red, which is compatible with green/yellow fluorescent reporters. However, both FM4–64 and PI have a number of limitations. First, they strongly label external cell/tissue layers but provide little or no labeling of internal layers. For example, in the root, the Casparian strip forms an impermeable barrier, which restrict the diffusion of FM4–64 and PI in internal tissues (i.e. the stele; Alassimone et al., 2010). Second, they wash away and bleach over time, which is problematic when performing long time-lapse acquisitions. In this case, they must be regularly reapplied to the mounting medium, which is not always convenient and can lead to variation in labeling intensities (Doumane et al., 2017). Third, FM4–64 becomes internalized through endocytosis overtime. This is actually a property of this dye that is often used to study endocytic processes (Rigal et al., 2015). However, strong labeling of intracellular compartments can be problematic for the automatic segmentation of cells.

As an alternative to FM4–64 labeling, transgenic lines stably expressing fluorescently tagged plasma membrane proteins can be used. One of the most widely used proteins is LOW TEMPERATURE-INDUCED PROTEIN 6B (Lti6b, also called RARE-COLD-INDUCIBLE 2B/RCI2b/At3g05890) and its tandem duplicated gene Lti6a/RCI2A (At3g05880; Figure 1B;  Kim et al., 2021). These two proteins were initially identified by Sean Cutler and colleagues in a screen for GFP-tagged proteins with interesting localizations. The corresponding transgenic lines are sometimes referred to as 29-1 and 37-26, which are the numbers of the original transgenic lines identified in this screen (Table 3; Cutler et al., 2000). Red and yellow variants are now available as well, increasing the palette of available transgenic lines (Elsayad et al., 2016; Noack et al., 2021). Other proteins that are often used as plasma membrane markers include aquaporins such as PIP2;1/PIP2a (also initially identified in Cutler et al. (2000) as line Q8) or PIP1;4 (Cutler et al., 2000; von Wangenheim et al., 2016), the formin FH6 (De Rybel et al., 2010), syntaxins such as SYP122 or NPSN12 (Assaad et al., 2004; Geldner et al., 2009; Vermeer et al., 2014; Barberon et al., 2016), lipid-anchored fluorescent proteins (e.g. myristoylation, acylation, prenylation; Vermeer et al., 2004; Simon et al., 2016; Willis et al., 2016; Yang et al., 2021), or lipid binding proteins (Simon et al., 2014, 2016; Table 3). Genetically encoded fluorescent plasma membrane markers avoid some but not all of the above-mentioned drawbacks of FM4–64. For example, it is not always easy to obtain a strict plasma membrane localization. Indeed, transmembrane proteins traffic through the endomembrane system to reach the plasma membrane and are degraded in the vacuole. This can be problematic for pH resistant fluorescent proteins (e.g. mCHERRY, mCITRINE) that are sometimes prominently seen in the vacuoles in some cell types or under certain growth conditions (e.g. lower pH of the vacuole in the dark). Extrinsic proteins may partition between the plasma membrane and the cytosol, which can affect segmentation. Other drawbacks of such reporter lines include (1) the bleaching of fluorescent proteins when imaged at high frequency rates, (2) the requirement for transgenesis, which may not be possible when studying certain species, and (3) the need to cross into the desired genetic background prior to imaging, which is time consuming.

**Table 3:** Fluorescent plasma membrane markers commonly used to label and segment cell contours in *A. thaliana*

PM Marker	PM targeting	Number of amino acids	Topology/ orientation	Comments	Ref. of transgenic line	NASC Stock #
Lti6b (RCI2b/29-1)	2 TM	54	Both termini are oriented toward the cytosol	From Ehrhardt GFP-fusion line collection	Cutler et al., 2000	N84726
Lti6a (RCI2a/37-26)	2 TM	54	Both termini are oriented toward the cytosol	From Ehrhardt GFP-fusion line collection	Cutler et al., 2000	N84758
PIP2;1 (PIP2a)	6 TM	287	Both termini are oriented toward the cytosol	From Ehrhardt GFP-fusion line collection	Cutler et al., 2000	N84725
PIP1;4 (W138)	6 TM	287	Both termini are oriented toward the cytosol	From wave line collection	Geldner et al., 2009	N781666; N781687; N781708
NPSN12 (W131)	1 TM	265	N-terminus in the cytosol	From wave line collection	Geldner et al., 2009	N781665; N781686; N781707
SYP122	1 TM	341	N-terminus in the cytosol		Assaad et al., 2004	–
FH6	1 TM	899	C-terminus in the cytosol		De Rybel et al., 2010	–
KA1	Anionic lipid binding	50	Extrinsic protein translated in the cytosol	KA1 domain of human MARK1 protein	Simon et al., 2016	N2107345
Myr	Lipid anchor: myristoylation	8	Facing the cytosol	First 8 aa of LeCPK1 must be located at the N-terminus	Willis et al., 2016	–
MAP (MP)	Lipid anchor: myristoylation and palmytoylation	12	Facing the cytosol	First 12 aa of AtGPA1 must be located at the N-terminus	Martinière et al., 2012	–
8K-Farn	Lipid anchor + anionic lipid biding: prenylation + cationic peptide	18	Facing the cytosol	Last 18 aa of human K-Ras4B, must be located at the C-terminus	Simon et al., 2016	N2017343
GPI	Lipid anchor: glycosylphos phatidylinositol	87	Facing the apoplast	aa 318–405 of AtAGP4, must be located at the C-terminus	Martinière et al., 2012	–

PM, plasma membrane, TM, transmembrane region. Lti6, LOW TEMPERATURE-INDUCIBLE; RCI, RARE-COLD INDUCIBLE; PIP, PLASMA MEMBRANE INTRINSIC PROTEIN; NPSN, NOVEL PLANT SNARE; SYP, SYNTAXIN OF PLANT; FH, FORMIN HOMOLOGY; KA1, KINASE-ASSOCIATED domain.

Once the plasma membrane (or alternatively the cell wall) is labeled with sufficient contrast, several software programs/algorithms have been developed to allow automatic extraction of cell contours, plant cell segmentation, and lineage tracing, including MorphographX, MARS/ALT, PlantSeg, and SurfCut (Fernandez et al., 2010; de Reuille et al., 2015; Erguvan et al., 2019; Strauss et al., 2019; Wolny et al., 2020; see Figure 1B for an example of segmentation using the level set method (LSM) (Kiss et al., 2017) and MorphographX (de Reuille et al., 2015)). Importantly, the plasma membrane is not a uniform compartment but is instead made up of a mosaic of small domains that are referred to as microdomains (>1 µm) or nanodomains (<1 µm) (Ott, 2017; Jaillais and Ott, 2020). Microdomains include polar domains within plant cells (see Ramalho et al. (2021) for a comprehensive review on the topic) as well as plant–microbial interfaces (Ott, 2017). Nanodomains are by definition small, and often their size is below the diffraction limit of optical microscopy. Several techniques have been used to visualize nanodomains in the living plant plasma membrane and to probe their dynamics, notably total internal resonance fluorescence microscopy (TIRFM), photoactivated localization microscopy (PALM), and single particle tracking techniques (Table 1; Martinière et al., 2012; Hosy et al., 2015; Gronnier et al., 2017; Wang et al., 2018; Martinière et al., 2019; Platre et al., 2019; Zhang et al., 2019; Smokvarska et al., 2020; Bayle et al., 2021; Noack et al., 2021). These methods have revealed a number of nanodomain-resident proteins, such a Remorins, Flotilins, HYPERSENSITIVE-INDUCED REACTION proteins, and receptor-like kinases (Li et al., 2012; Bucherl et al., 2017; Daněk et al., 2020; Gronnier et al., 2020; Jaillais and Ott, 2020; Gouguet et al., 2021; Martinière and Zelazny, 2021), as well as some proteins with a dynamic association with nanodomains, such as small GTPases from the RHO-OF-PLANTs family (Platre et al., 2019; Smokvarska et al., 2020, 2021; Bayle et al., 2021; Fuchs et al., 2021). Both microdomains and nanodomains not only have a specific protein composition but also accumulate specific lipid species (see “Imaging lipids” below) and are highly interconnected with the rest of the endomembrane network via both the vesicular and nonvesicular transport of materials.

### Imaging intracellular trafficking, fast and tiny!

The plasma membrane is part of the endomembrane system, a network of membranes interlinked by vesicular trafficking and direct membrane contacts (Boutté and Jaillais, 2020). This system includes the endoplasmic reticulum and the connected nuclear envelope, the Golgi apparatus and trans-Golgi Network (TGN), endosomes, vacuoles, and lysosomes, and the plasma membrane (Boutté and Jaillais, 2020). A number of dyes label specific parts of the endomembrane network. As mentioned above, FM4–64 is a prominent tool used to study the dynamics of endocytic processes because it can be used in pulse-chase experiments (Rigal et al., 2015; Johnson et al., 2020). Depending on the timing following FM4–64 treatment, it can either label (1) the plasma membrane specifically, (2) the plasma membrane and early endosomes/TGN, or (3) the plasma membrane, early and late endosomes, and the tonoplast (Dettmer et al., 2006; Jaillais et al., 2006, 2008; Geldner et al., 2009; Rigal et al., 2015). There are also dyes that label the vacuole, such as 2′,7′-Bis-[2-Carboxyethyl]-5-[and-6]-Carboxyfluorescein (BCECF) (Scheuring et al., 2016; Takemoto et al., 2018). Combined with fluorescence recovery after photobleaching (Table 1), BCECF allowed the connection between vacuoles within cells to be studied (Scheuring et al., 2016).

For the most part, plant cell biologists use fluorescent fusions with proteins targeted to specific compartments. The number of such fluorescent markers exploded since the publication of the Cutler collection, which initially identified markers for many cellular compartments (Cutler et al., 2000). In addition, a landmark resource in terms of endomembrane markers is the Waveline collection, which not only provided multiple markers for each compartment, but did so in several colors (Geldner et al., 2009). Having markers of different colors is critical for colocalization experiments. Indeed, most intracellular compartments seen under a confocal microscope look like dots and cannot be irrefutably identified based on their morphology alone. The sensitivity to drugs can be used to discriminate between different membrane compartments (Geldner et al., 2003; Dettmer et al., 2006; Jaillais et al., 2006, 2008; Worden et al., 2014; Kania et al., 2018; Mishev et al., 2018), but colocalization is the gold standard. Importantly, as discussed above for plasma membrane proteins, strict localization in a single compartment is very rare. To obtain a robust idea of the localization of a given protein, it is thus essential to perform quantitative colocalization with many different markers. Quantification of colocalization can be tricky; several methods for doing this are described in some excellent reviews (Bolte and Cordelieres, 2006; Lagache et al., 2015, 2018; Aaron et al., 2018).

There are two major difficulties when studying the dynamics of the endomembrane system. First, vesicles and membrane domains are often tiny, being at or below the optical resolution of light microscopy (∼250 nm; Sahl et al., 2017; Schermelleh et al., 2019). Second, membrane trafficking is fast, with certain compartments moving tens of micrometers per minute, notably due to cytoplasmic streaming (Luo et al., 2015). In terms of resolution, there are more and more examples of super-resolution microscopy methods used in plants (Komis et al., 2015a, 2015b; Schubert, 2017; Shaw et al., 2019; Bayle et al., 2021). These methods can provide large gains in resolution, such as PALM (Hosy et al., 2015; Gronnier et al., 2017; Martinière et al., 2019; Platre et al., 2019; Bayle et al., 2021) and stimulated emission depletion microscopy (Kleine-Vehn et al., 2011; Demir et al., 2013) or provide ultrafast high-resolution imaging, such as super-resolution confocal live imaging microscopy (SCLIM) (Table 1; Naramoto et al., 2014; Uemura et al., 2014, 2019; Shimizu et al., 2021). SCLIM in particular appears to be well suited to study membrane trafficking events in plants. For example, three-colored 4D imaging of the Golgi and the TGN was recently reported in Arabidopsis roots, allowing highly specialized subdomains within the TGN to be identified (Shimizu et al., 2021).

To image events that occur at or close to the plasma membrane, the technique of choice is TIRFM (or derivatives of the TIRF technique such as variable angle epifluorescence microscopy) , which is a very sensitive technique because it does not collect any out-of-focus light (Table 1; Konopka et al., 2008; Konopka and Bednarek, 2008; Gronnier et al., 2017; Johnson and Vert, 2017; Platre et al., 2019; Johnson et al., 2020; Narasimhan et al., 2020; Smokvarska et al., 2020; Bayle et al., 2021). TIRFM has mainly been used to study endocytosis, but also cellulose synthesis and cytoskeleton dynamics, and it can be combined with structured illumination microscopy to achieve fast super-resolved acquisition (Table 1; Johnson et al., 2021). Quantification methods to study endocytosis in plants were recently reviewed (Dragwidge and Van Damme, 2020; Johnson et al., 2020).

### Imaging lipids

**Figure 2:**
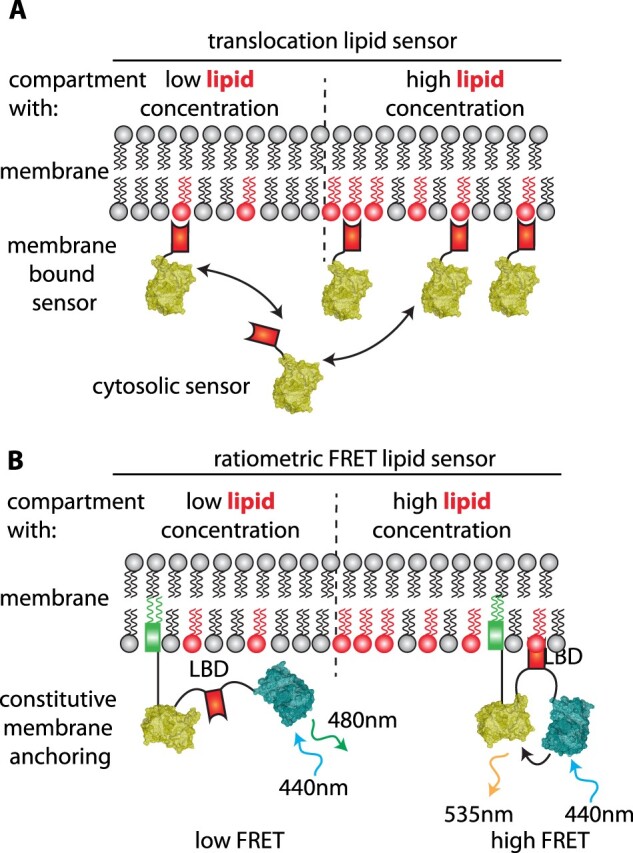
Principles of genetically encoded lipid biosensors. A, Schematic representation of “translocation” lipid sensors. Their localization alternates between membrane-bound and cytosolic. Their membrane-bound fraction increases with increasing concentration of lipids, but this can be difficult to quantify. B, Schematic representation of ratiometric FRET-based lipid sensors, such as PAleon. They are more quantitative than translocation sensors, but are constitutively targeted to a predetermined membrane. They can thus be used once the membrane of interest has been identified (e.g. using translocation sensors).

Unlike proteins, membrane lipids cannot be genetically tagged with a fluorescent protein. While it is possible to use cellular fractionation or immunolocalization, these techniques are not amenable to live samples. In contrast, genetically encoded biosensors are compatible with live imaging (Platre and Jaillais, 2016). In their simplest form, lipid biosensors are sometimes referred to as translocation sensors (Platre and Jaillais, 2016; Wills et al., 2018). They consist of an isolated lipid binding domain known to interact stereo-specifically with a given lipid species fused with a fluorescent protein (Figure 2A). These domains are generated in the cytosol and are targeted to the membranes *via* interaction with their cognate lipids, hence the term “translocation sensors”. These sensors were instrumental in studying the subcellular accumulation of lipids and helped draw a map of lipid localization in plant cells (Table 4; Vincent et al., 2005; Vermeer et al., 2006, 2009, 2017; van Leeuwen et al., 2007; Simon et al., 2014, 2016; Hirano et al., 2017, 2018; Noack and Jaillais, 2017; Platre et al., 2018; Noack and Jaillais, 2020; Xing et al., 2021; Ito et al., 2021). However, like any genetically encoded biosensors, they have inherent caveats including (1) competition with endogenous lipid binding proteins, (2) potential masking of their endogenous ligands, and (3) the fact that these lipid binding domains usually rely on additional membrane features for localization (Heilmann, 2016; Platre and Jaillais, 2016; Dubois and Jaillais, 2021). In addition, they mostly recognize the lipid head groups and, in fact, are available only to study anionic phospholipids. Indeed, no biosensors for abundant structural phospholipids, sterols, or sphingolipids have been characterized to date. This is mostly due to the lack of known lipid binding domains with specific binding to these lipids.

**Table 4:** Commonly used anionic lipid sensors in *A. thaliana*

Lipid	Sensor name	Sensor type	Localization in root tip	Comments	Ref. of transgenic line	NASC Stock #
PI3P	PX^p40^ (P3)	Translocation	Late endosome/tonoplast		Simon et al., 2014	N2105606; N2105615; N2105623
	2xFYVE^HRS^ (P18)	Translocation	Late endosome/tonoplast		Vermeer et al., 2006, Simon et al., 2014	N2105611; N2105620; N2105626
PI4P	1xPH^FAPP1^ (P5)	Translocation	PM (++)/TGN (+)/cell plate (+++)	Coincident detection of PI4P and ARF1	Vermeer et al., 2009, Simon et al., 2014	N2105607; N2105616; N2106624
	2xPH^FAPP1^ (P21)	Translocation	PM (+++)/weak TGN/cell plate (+++)	High affinity sensor	Simon et al., 2014	N2105612; N2105621
	3xPH^FAPP1^	Translocation	PM (+++)/occasional TGN/cell plate (+++)	High affinity sensor	Simon et al., 2016	–
	1xPH^FAPP1-E50A^	Translocation	PM (+++)/occasional TGN/cell plate (+++)	ARF1-binding site mutated	Ito et al., 2021	–
	P4M^SiDM^	Translocation	PM/cell plate (+++)		Simon et al., 2016	N2017346
PI(4,5)P_2_	1xPH^PLC^ (P14)	Translocation	Weak PM/cytosol	Low affinity	Vincent et al., 2005, van Leeuwen et al., 2007, Simon et al., 2014	N2105609; N2105618; N2105625
	2xPH^PLC^ (P24)	Translocation	PM/cytosol	High affinity	Simon et al., 2014	N2105613; N2105622
	TUBBY-C (P15)	Translocation	PM/cytosol + nucleus		Simon et al., 2014	N2105610; N2105619
PI(3,5)P_2_	2xML1N	Translocation	Late endosome (≠PI3P endosome)		Hirano et al., 2017	–
PA	1xPASS	Translocation	Weak PM/cell plate		Platre et al., 2018	N2107781
	2xPASS	Translocation	PM/cell plate/nucleus	High affinity	Platre et al., 2018	N2107782
	PAleon	FRET, ratiometric	Constitutive targeting at PM	Ratiometric / quantitative	Li et al., 2019a, 2019b	–
PS	C2^Lact^	Translocation	PM/cell plate/endosomes/tonoplast		Simon et al., 2016; Platre et al., 2018	N2117347; N2107778
	2xPH^EVCT2^	Translocation	PM/cell plate/endosomes/tonoplast		Platre et al., 2018	N2107779; N2107780
DAG	1xC1a^PKC^	Translocation	Mostly cytosolic/PM/cell plate/TGN		Vermeer et al., 2017	–
	2xC1a^PKC^	Translocation	Cytosol/PM/cell plate/TGN	High affinity	Vermeer et al., 2017	–

FAPP1, four-phosphate-adaptor protein 1; HRS, hepatocyte growth factor-regulated tyrosine kinase substrate; PLC, phospholipase C; ML1N, cytosolic phosphoinositide-interacting domain (ML1N) of the mammalian lysosomal transient receptor potential cation channel, Mucolipin 1 (TRPML1); PASS, PA biosensor with superior sensitivity; Lact, lactadherin; EVCT2, EVECTIN2; PKC, protein kinase C.

Because these sensors are produced in the cytosol, they are designed to study the lipid embedded in the cytosolic leaflets, not the extracellular or luminal membrane leaflet, which is an additional limitation of these sensors (Figure 2A). Finally, because they are based on translocation, which can be tricky to quantify, these sensors are useful for studying the subcellular localization of anionic lipids, but are of limited interest for studying the amounts of lipids in different cells or tissues (Colin and Jaillais, 2019). Quantification of the relative levels of lipids can be achieved using fluorescence resonance energy transfer (FRET)-based lipid sensors (Figure 2B;  Platre and Jaillais, 2016). To date, there is only one such sensor available in plants for phosphatidic acid (PA; Li et al., 2019b). This ratiometric sensor, named PAleon, is based on a PA-binding domain, which is sandwiched between two fluorescent proteins (a FRET donor and acceptor, Table 1) and constitutively anchored to the plasma membrane (Li et al., 2019b; Mamode Cassim and Mongrand, 2019). PA-binding triggers a conformational change in the sensor, which decreases the distance between the acceptor and donor fluorescent proteins and thus a change in FRET (Figure 2B). Using PAleon, PA levels were shown to rapidly change upon abiotic stress, which was known from previous biochemical studies. In addition, these changes are highly tissue specific in the root, a feature that could not be addressed using traditional biochemical approaches (Li et al., 2019b). FRET-based sensors for other lipids have been used in animal cells (Platre and Jaillais, 2016; Wills et al., 2018) and are eagerly awaited for studying and quantifying the levels of other lipids in plants. Other approaches that could complement the biosensor approaches are based on in vivo lipid labeling, for example via click-chemistry (Neef and Schultz, 2009; Tamura et al., 2020). These approaches are starting to be available for plant samples (Paper et al., 2018), but as far as we know, they have not yet been used on live plant tissues. It is also possible to use exogenous treatments with fluorescently labeled lipids (Poulsen et al., 2015; Zhang et al., 2020b; Susila et al., 2021), but it might be tricky to assess whether the localization of exogenously added lipids reflects the true localization of endogenous lipids (in terms of subcellular accumulation, leaflet association, and potential degradation of the fluorescent lipid; Grabski et al., 1993).

## Live imaging of plant hormones

### Imaging the transcriptional output of hormones

**Figure 3:**
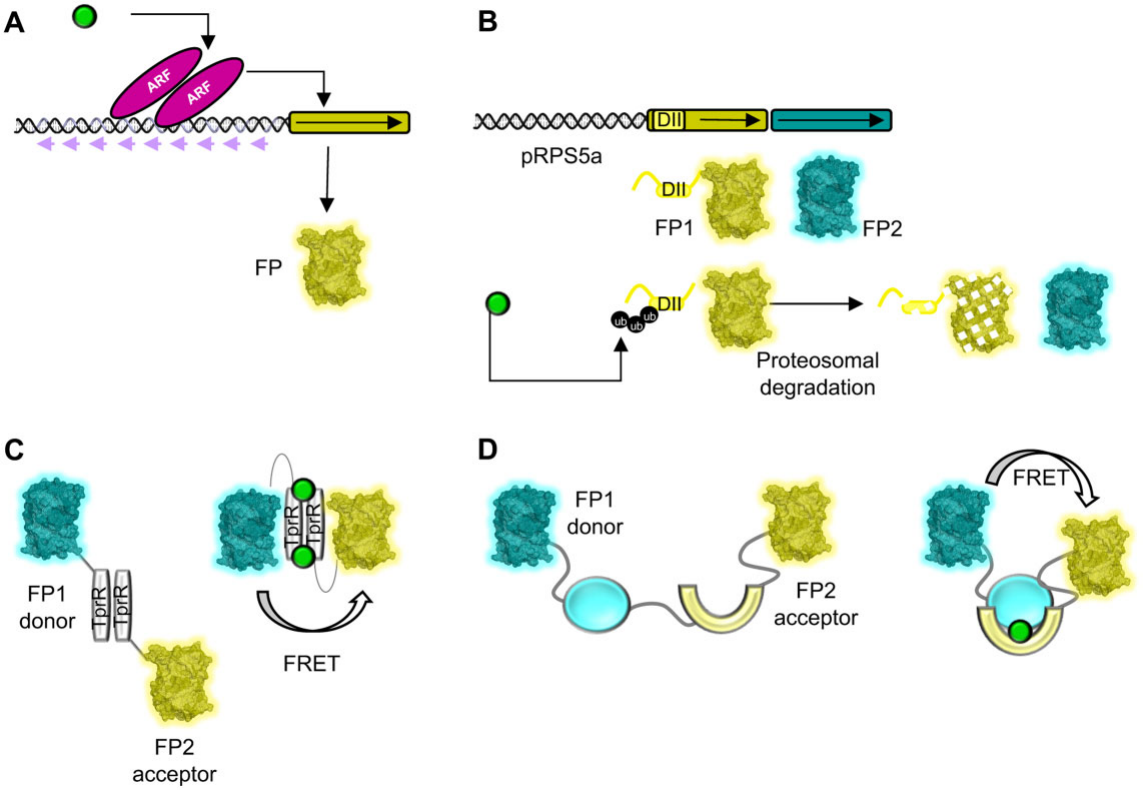
Design principles of different types of plant hormone sensors. A, DR5, an example of a plant hormone transcriptional sensor. The DR5 auxin synthetic promoter contains nine repeats (violet arrows) of ARF TF binding sites that control the expression of a FP in response to the hormone (green circle). B, qDII, an example of a plant hormone degradation-based sensor. The qDII ratiometric sensor is composed of two FPs: FP1, fused to a DII degron domain; and FP2, whose expression is controlled by the same constitutive promoter. Auxin triggers ubiquitination of the DII domain and the further degradation of FP1. This can be quantified using the FP2 signal, which remains constant, as a reference. C, D, FRET-based plant hormone sensors. Two types of FRET sensors are available. The auxin FRET sensor AuxSen (C) uses the dimer of TprR (*Escherichia coli* tryptophan repressor, in grey) fused to two FPs, the donor and acceptor, which come in close contact due to a conformational change that follows auxin binding to TprR. For ABACUS, ABAleon (ABA), and GPS1 (GA) FRET sensors (D), donor and acceptor FPs are fused to two protein interacting partners (light blue and yellow) that bind to each other in the presence of the hormone.

Recent years have seen an explosion in the number of genetically encoded biosensors, mainly developed in Arabidopsis, to detect hormones at high spatio-temporal resolution within living tissues using fluorescence microscopy (for recent reviews and a more exhaustive discussion on genetically encoded biosensors, see: Walia et al. (2018), Martin-Arevalillo and Vernoux (2019), and Isoda et al. (2021)). Here, we highlight the most commonly used of these biosensors (Table 5). A pioneering work that initiated these developments is the construction of the DR5 auxin transcriptional sensor (Ulmasov et al., 1997, 1999; Sabatini et al., 1999; Benkova et al., 2003; Ottenschlager et al., 2003) and its more recent derivative DR5v2 (Liao et al., 2015). Both consist of a synthetic auxin-responsive promoter, with multiple binding sites for auxin response factors, driving the expression of a fluorescent protein (FP; Figure 3A). Indeed, plant hormones regulate gene expression via transcription factors specific to each pathway that recognizes specific binding sites (Larrieu and Vernoux, 2015). Strategies similar to the one used for DR5 were then leveraged to design transcriptional biosensors for cytokinins (Müller and Sheen, 2008; Zürcher et al., 2013; Steiner et al., 2020), ethylene (Stepanova et al., 2007), and abscisic acid (ABA; Table 5; Wu et al., 2018). While endogenous promoters of hormone-responsive genes have also been used to analyze the transcriptional responses to hormones, the use of synthetic promoters increases the specificity of the response of the biosensor to a given hormone. Transcriptional biosensors have provided invaluable data on the physiology and roles of these four plant hormones in development (Isoda et al., 2021). Importantly, hormone signaling pathways include feedback mechanisms and thus have nonlinear topologies. A classic example is the auxin pathway, which includes negative feedback (Weijers and Wagner, 2016). Therefore, transcriptional biosensors of hormones do not have an activity that is linearly dependent upon hormone levels. Instead, they provide information on the processing properties of the signaling pathway that functions downstream of the hormone. In addition, the activity of the transcription factors controlling the expression of transcriptional biosensors might be regulated by other signals. Thus, transcriptional biosensors can also be influenced by crosstalk between pathways (Nemhauser et al., 2004; Jaillais and Chory, 2010).

**Figure 4:**
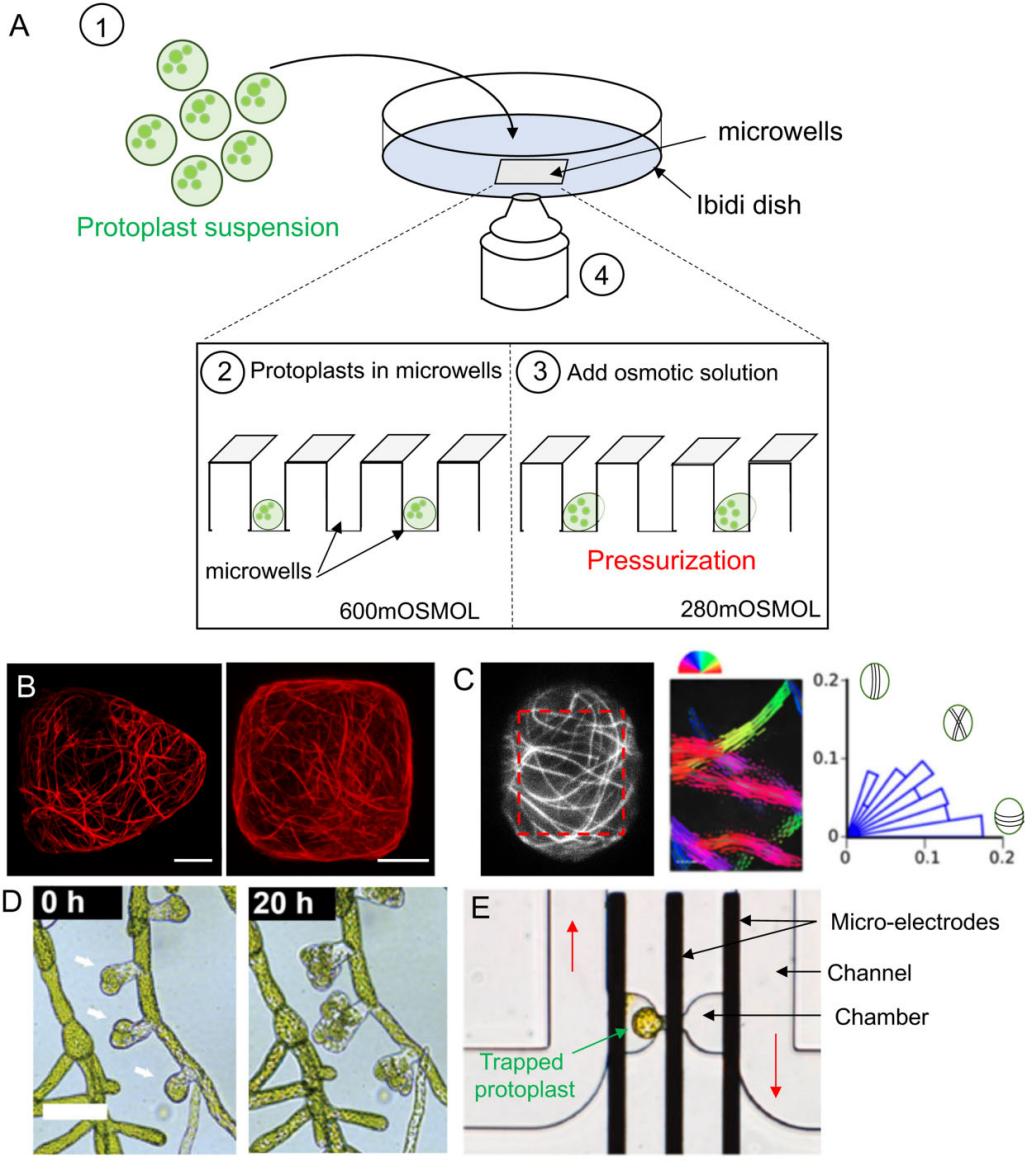
Single-cell approaches to study cellular responses to mechanical forces. A, Schematic representation of the device used to confine protoplasts to microwells (adapted from Colin et al., 2020). Briefly, a drop of a solution containing a suspension of protoplasts is deposited into the microwells of an Ibidi dish (1). Close-up of microwells containing protoplasts in 600 mOSMOL mannitol solution (2). Once in microwells, the protoplasts are ready to be imaged (4). In this figure, protoplasts are pressurized using a hypo-osmotic solution (280 mOsmol mannitol (3), as in Colin et al., 2020). Many other types of experiments can be done (microwell coating, cell division experiments, and so on). B, Microtubule signals (P35S:GFP-MBD) in deformed protoplasts confined in agar wells (adapted from Durand-Smet et al., 2020). Scale bar, 10 µm. C, Analysis of microtubule orientation (adapted from Colin et al., 2020). Example of microtubule signal (p35S:GFP-MBD) in a protoplast confined in a 15 × 20-µm microwell. The doted red line represents the ROI in which cortical microtubule orientation has been performed (left). The orientation of cortical microtubules in each ROI is color coded (middle). Polar histograms represent the cortical microtubule angle distribution for the protoplast (right). Each bar corresponds to an angle range of 9°. Schematic representation of cortical microtubule orientations are indicated on the plot. D, Time-lapse recording of the development of leafy buds of *Physcomitrium patens* (adapted from Sakai et al., 2019). Arrows indicate leafy buds. Scale bar, 70 µm. E, Microscope image of a trapped Arabidopsis mesophyll cell (adapted from Chen et al., 2020). Flow direction was from left to right (red arrows). The three coplanar microelectrodes are represented by parallel black thick lines. The middle electrode acts as the exciting electrode.

**Table 5:** Genetically encoded hormone sensors available as stable *A. thaliana* transgenic lines

Hormone	Sensor name	Sensor type	Comments	Ref. of transgenic line	NASC Stock #
Auxin	DR5	Transcriptional	Nine inverted repeats of *TGTCTC*	Ulmasov et al., 1997	N9402, N9361, N799364, N2106112, N2106143, N2106173
	DR5v2	Transcriptional	Nine inverted repeats of *TGTCGG*	Liao et al., 2015	N2105636
	DII-VENUS	Degradation	Domain II of IAA28 fused to fast-maturing yellow fluorescent protein VENUS	Brunoud et al., 2012	N799173
	R2D2	Degradation, ratiometric	Ratiometric expression of DII-3xVENUS and mDII-ntdTOMATO from two RPS5A promotors	Liao et al., 2015	N2105637
	qD2	Degradation, ratiometric	Ratiometric expression of DII-VENUS and TagBFP from a single RPS5A promotor	Galvan-Ampudia et al., 2020	–
	AuxSen	FRET, ratiometric	Engineering of tryptophan sensor to recognize auxin	Herud-Sikimic et al., 2021	N2110798–N2110801
GA	RGA^mPFYR^	Degradation, ratiometric	GA-responsive DELLA without its regulatory function in transcriptional response	Shi et al., 2021	–
	GPS1	FRET, ratiometric	Based on GID1/GAI interaction	Rizza et al., 2017	–
ABA	6xABRE-R	Transcriptional	6xABRE element from *RD29A*	Wu et al., 2018	N71620
	6xABRE-A	Transcriptional	6xABRE element from *ABI1*	Wu et al., 2018	N71619
	ABACUS	FRET, ratiometric	Based on PYL1/ABI interaction	Jones et al., 2014	–
	ABAleon	FRET, ratiometric	Based on PYR1/ABI1 interaction	Waadt et al., 2014, 2020	–
	SNACS	FRET, ratiometric	Sensors of OST1/SnRK2.6 activity, based on 14-3-3/AKS1 interaction	Zhang et al., 2020a, 2020b	–
CK	TCS	Transcriptional	Six direct repeats of type B ARR-binding (A/G)GAT(T/C) element	Müller and Sheen 2008	N69181, N23900 N66322
	TCSn	Transcriptional	Tandem head-to-head and tail-to-tail orientations of type B ARR-binding (A/G)GAT(T/C) element	Zürcher et al., 2013	N69180
	TCSv2	Transcriptional	Alternating head-to- head and tail-to-tail orientations of type B ARR-binding (A/G)GAT(T/C) element	Steiner et al., 2020	–
JA	Jas9-VENUS	Degradation	Jas domain of JAZ9 fused to the fast maturing VENUS-N7	Larrieu et al., 2015	N2105629
SLs	Strigo-D2	Degradation	Truncated domain of AtSMXL6 (aa 615–979) fused to fast maturing mVENUS	Song et al., 2021	–

Note that we referenced only reporters that have been engineered to act as biosensors in the sense that they represent minimal systems to report on hormonal activities. We thus excluded from this table full-length hormone-responsive promoters or proteins (that can be degraded or change localization upon hormone signaling), since they are more likely to be regulated by additional cues and to modify the system they are supposed to monitor.

### Measuring hormonal input

Understanding how a given hormone regulates transcriptional responses within a tissue requires direct information about the distribution of the hormone to be obtained. Two complementary strategies have been used in parallel to tackle this challenge. The first strategy is based on the observation that several hormones (auxin, jasmonates, gibberellins [GA], ABA, salicylic acid, strigolactones, and karrikin) trigger rapid degradation of signaling effectors through polyubiquitination by the Skp-Cullin-F-box complex (Larrieu and Vernoux, 2015). This has led to the design of degradation-based biosensors. This strategy was first implemented for auxin with the development of the DII-VENUS biosensor (Brunoud et al., 2012) and its ratiometric versions R2D2 and qDII (Liao et al., 2015; Galvan-Ampudia et al., 2020; Figure 3B and Table 5). The level of the DII-VENUS synthetic protein is inversely correlated to the concentration of auxin across a large range of concentrations, allowing auxin distribution to be mapped at cellular resolution during development (for a specific review on this subject, see Martin-Arevalillo and Vernoux (2019). This auxin degradation-based biosensor was shown to function in Arabidopsis and in a variety of other plants such as maize (Mir et al., 2017), Brachypodium (O'Connor et al., 2017), and more recently mosses (Landberg et al., 2021), demonstrating the wide applicability of this design to evolutionarily distant plants. Synthetic degradation-based biosensors have also been generated for jasmonates (Jas9-VENUS; Larrieu et al., 2015), GA (qRGA^mPFYR^; Shi et al., 2021), and strigolactones (Strigo-D2; Song et al., 2021) using a ratiometric design. While degradation-based biosensors have proven to be powerful and easy-to-use tools to analyze hormone contents in living tissues, they also have a number of limitations. The detection remains indirect, as degradation of the biosensor uses the hormone perception cellular machinery, which can induce detection biases, for example upon differential expression of receptors (Vernoux et al., 2011; Brunoud et al., 2012). In addition, their spatial definition is limited to the cellular scale or above, and they cannot detect rapid variations in hormone levels, as they need to be re-synthesized following degradation.

The design of direct biosensors (i.e. biosensors that autonomously detect hormones) is a second strategy that has been used in a handful of studies to detect hormone distribution even below the cellular scale. FRET biosensors have been developed for ABA (ABACUS, ABAleon, SNACS; Jones et al., 2014; Waadt et al., 2014; Zhang et al., 2020a), GA (GPS1; Rizza et al., 2017), and more recently auxin (AuxSen; Herud-Sikimic et al., 2021; Table 5). FRET biosensors use two FPs and the physical property of a donor FP excited at a certain wavelength to transfer energy to an acceptor FP that will then fluoresce (Table 1). Here, this energy transfer is modified by the binding of the hormone (Figure 3, C and D). The FRET biosensors allow for the rapid, quantitative detection of hormones within living tissues in Arabidopsis, where they have been tested so far, and have been used to follow the hormone distribution dynamics during developmental processes and environmental responses (Jones et al., 2014; Waadt et al., 2014, 2020; Rizza et al., 2017; Herud-Sikimic et al., 2021). While both the ABA and auxin FRET biosensors have been shown to function in different intracellular compartments (Jones et al., 2014; Herud-Sikimic et al., 2021), FRET biosensors are yet to be used to analyze hormone distribution in different cell compartments or in the apoplast. This is notably, but certainly not exclusively, a key missing piece of information for auxin given that multiple intracellular transporters regulate auxin responses (Sauer and Kleine-Vehn, 2019). FRET biosensors are not without limitations. Notably, ABACUS, ABAleon, and GPS1 expression leads to hypersensitivity to their hormone target. The FRET activity of GPS1 is also partly reversible, and the range of concentrations detected by the existing FRET sensors might not cover the entire range of endogenous concentrations (for an exhaustive comparison, see Isoda et al. (2021)). Further optimization will certainly allow these limitations to be minimized (Waadt et al., 2020) or even eliminated.

### Using quantitative live imaging to understand hormonal processing

The different types of biosensors currently available provide a powerful toolbox to bring our knowledge of hormone action during developmental and environmental responses to the next level, from the cellular scale to the plant scale and even the population scale. Such technology opens up extensive possibilities. For example, transcriptional and degradation-based/FRET biosensors could be combined to understand how hormonal signals are dynamically processed by signaling pathways to induce downstream changes in gene expression in living tissues. This has been done for auxin by combining DR5 and DII-VENUS or qDII biosensors, revealing differences in auxin sensitivity between functional domains of the shoot apical meristem and the requirement for sustained exposure to high auxin levels for the induction of transcription (Vernoux et al., 2011; Ma et al., 2019; Galvan-Ampudia et al., 2020). Biosensors for different hormones could also be combined to understand their respective contributions to developmental and environmental responses, such as for auxin and cytokinins, which often act antagonistically. A large number of FRET sensors are also available for detecting endogenous metabolites and small molecules (for review, see Walia et al. (2018) and Isoda et al. (2021), which could be combined with hormone biosensors. This was recently done with ABA FRET biosensors and biosensors for Ca^2+^, protons, chloride, H_2_O_2_, and glutathione redox potential (Walia et al., 2018; Waadt et al., 2020). This study showcased how the effects of hormones on key secondary messengers can be followed at high spatio-temporal resolution, demonstrating (for example) that GA does not trigger rapid changes in pH or Ca^2+^. We expect that this toolbox will continue to be developed in the near future. For example, sensors for strigolactones have been tested in protoplasts (Samodelov et al., 2016; Chesterfield et al., 2020; Braguy et al., 2021) and are now emerging in planta (Song et al., 2021). More such sensors will certainly emerge.

## Live imaging of the mechanical properties of cells and tissues and their responses to forces

In the last decades, biophysical approaches have been developed to probe the mechanical properties of plant cells and their response to forces. Below, we review how live imaging has taken on central importance in the emergence of this field of cell and developmental biology.

### Atomic force microscopy: probing for cell mechanical properties

Atomic force microscopy (AFM) belongs to the family of scanning probe microscopy techniques, where a tip (or probe, usually with a nanometric radius) scans the surface of a sample (Binnig et al., 1986). While in the case of optical or electron microscopies, topographic information about the sample is gathered using the transmission or reflection of a beam, in the case of AFM, it is the interaction force between the tip and the sample surface that is used. In the case of contact mode operation, for example, the tip scans the surface while the system monitors the tip-sample force and acts to maintain it at a constant level: if the sample surface is not atomically flat and perfectly horizontal (i.e. lying on the xy scanner’s plane), the tip has to be moved up and down to maintain the force unchanged. Those displacements are then collected to reconstruct a 3D topography of the surface. Depending on the tip used and the scanning conditions, lateral and vertical resolutions may be <1 nm. Since this type of microscope can easily be operated in liquid medium, its application in biology, particularly for living samples, is rather natural and advantageous compared to other microscopy techniques.

Beyond topography, AFM allows any type of interaction forces to be detected, such as electrostatic, van der Waals, or contact forces or specific interaction forces between the tip and the sample, down to few piconewtons. In addition, the tip can be used to apply forces at the surface of a sample while measuring the resulting deformation (indentation) in order to determine its mechanical properties (e.g. Young’s modulus, viscoelastic properties).

Understanding the role of plant cell wall mechanics is essential for explaining the mechanisms underlying developmental processes and morphogenesis (Hamant and Traas, 2010; Mirabet et al., 2011; Sapala et al., 2018; Landrein and Ingram, 2019; Vernoux et al., 2021). Indeed, along with genetic regulation and growth factors, the mechanical properties of the cell wall are tightly regulated: for example, cell wall softening is required to allow cell growth. AFM allows these properties to be measured and the way they change within/between organs, genotypes or developmental stages to be studied (Milani et al., 2011; Peaucelle et al., 2011; Yakubov et al., 2016). AFM can also be coupled with fluorescent microscopy to provide correlative information between the mechanical properties of a cell/tissue and the expression of marker genes (Milani et al., 2014). Elastic modulus maps can be generated by creating a series of force curves (where the tip is alternatively placed onto and withdrawn from the surface) on a matrix defined for a ROI in the sample (for advice on how to set up this type of experiment, see, for example, Bovio et al., 2019; Braybrook, 2015). These curves are often analyzed using standard contact models (e.g. Hertz, Sneddon), which can be used to calculate the elastic modulus per curve. More advanced measurements can also be set up to study the sample’s viscoelastic properties (for example in animal cells, see Alcaraz et al., 2003; Rother et al., 2014) or to evaluate cell turgor pressure at the single cell (Beauzamy et al., 2015; Long et al., 2020) or organismal level (Beauzamy et al., 2016; Creff et al., 2021).

Despite their versatility and high lateral resolution, scanning probe techniques are intrinsically limited to the study of the sample’s surface. More recently, new techniques known as optical or photo-acoustic elastographies (Larin and Sampson, 2017; Singh and Thomas, 2019) have been developed that provide information on the mechanical properties of the volume of a biological sample. One of these techniques is Brillouin microscopy, which uses Brillouin scattering to extract the longitudinal storage moduli of samples (Antonacci et al., 2020). This technique is based on the inelastic scattering of an incident photon by a phonon (pressure wave) of the sample. The process is similar to Raman scattering, but instead of modes of vibration of single molecules, the information is retrieved from propagating phonons, thus providing access to the mechanical properties of the material (Antonacci et al., 2020). This technique has already been applied to plant tissues (Elsayad et al., 2016). However, it is still in its early stage of development, meaning that the experimental set-up and analysis framework have to be optimized to provide specific and reliable information on the mechanical properties of biological samples.

### Measuring cellular responses to forces

Plant organs are exposed to specific patterns of mechanical forces that can be perceived by cells and influence key processes such as growth, division, polarity, and gene expression (Landrein and Ingram, 2019). At the single cell level, mechanical stress builds up from the hydrostatic pressure of the cell (i.e. turgor), which puts the surrounding walls under tension and induces growth when the yielding threshold of these walls is exceeded (Lockhart, 1965). At the organ level, mechanical stresses often build up from mechanical conflicts caused by differences in mechanical properties (pressure and wall properties) between cells and tissues (Kutschera and Niklas, 2007; Hamant et al., 2008). As stress patterns are of a purely physical nature, they can be predicted using mechanical models (Hamant et al., 2008; Heisler et al., 2010; Bozorg et al., 2014; Sampathkumar et al., 2014), but they cannot be directly measured easily. However, they can also be indirectly assessed by measuring the turgor pressure of the cell, the strain (i.e. deformation) they induce (notably at the membrane or in the cell wall), and the physiological response they trigger in the cell.

Cell turgor can be directly measured in living tissues using a pressure probe, but this method is invasive and difficult to use for small cells (Beauzamy et al., 2014). A less-invasive method has thus been developed in which turgor pressure values are extracted based on indentations generated with an AFM (Beauzamy et al., 2015). This technique was recently used to unravel differences in pressure between cells in the epidermis of the shoot apical meristem (Long et al., 2020). However, this method is indirect, as pressure information must be extracted from force measurements using physical models, and this method cannot be used to measure cell turgor in inner tissues. To overcome these limitations, a new FRET sensor was recently developed to directly probe cell osmolarity (Cuevas-Velazquez et al., 2021). This sensor is based on the use of an intrinsically disordered protein that is normally expressed under water deficit conditions, and whose structure depends on the osmolarity of the medium. Measuring the strain (elastic or plastic deformation) induced by mechanical forces on the cell, and notably at the membrane or within the cell wall, is challenging. Microviscosity sensors were recently developed to probe the mechanical environments of different cell compartments (Michels et al., 2020). These sensors are molecular rotors whose rate of intramolecular rotation, and thus their fluorescent lifetime (imaged by fluorescence lifetime imaging microscopy [FLIM], Table 1), depends on their mechanical environment. Microviscosity sensors have been used to unravel the existence of specific patterns of membrane and wall microviscosity in roots and pavement cells. These patterns have been linked to changes in membrane and wall composition but also to spatial and temporal variations in membrane and wall tension, notably in response to changes in turgor pressure. These microviscosity FLIM sensors thus appear to be unique tools for assessing mechanical stress patterns within plant organs (Michels et al., 2020).

Mechanical forces could also be visualized and measured based on the response they induce in the cell. The mechanisms through which cells are able to sense forces in plants are largely unknown. It has been hypothesized that mechanical stress could be perceived at the interface between the plasma membrane and the cell wall through receptor-like kinases (such as FERONIA) and/or through membrane-associated channels (such as OSCA, DEK1, or PIEZO; Landrein and Ingram, 2019; Codjoe et al., 2021; Fobis-Loisy and Jaillais, 2021). To our knowledge, fluorescent sensors derived from potential mechanosensors have not yet been developed. Alternatively, it has also been shown that cortical microtubules robustly respond to the application of mechanical forces in a variety of plant organs (Hamant et al., 2008; Sampathkumar et al., 2014; Robinson and Kuhlemeier, 2018) and it has even been hypothesized that microtubules themselves may act as mechanosensors (Hamant et al., 2019b). Mechanical forces can thus be probed by measuring the level of organization and orientation of cortical microtubules by confocal microscopy. This method has been applied to the seed, where the repartition of forces within the layers of the outer-integument (outermost layers of the seed coat) was assessed by comparing microtubule organization with the responses to forces within these layers (Creff et al., 2015). However, it is important to note that microtubules can also respond to other signals such as light and hormones; thus, their organization may not only be linked to mechanical stress patterns (Landrein and Hamant, 2013).

Mechanical forces can also be assessed by quantifying the expression of fluorescent reporters for mechanosensitive genes such as *ZINC FINGER PROTEIN2* in stems, *SHOOT MERISTEM LESS* in meristems, and *EUI-LIKE P450 A1* in seeds (Martin et al., 2014; Creff et al., 2015; Landrein et al., 2015). This approach was recently applied to developing seeds to assess stress levels in a mutant impaired in turgor pressure (Creff et al., 2021). However, this type of analysis is limited by the fact that the mechanosensitive genes that have been characterized to date are only expressed in a small subset of cells in specific tissues. Finally, mechanical perturbations have been shown to trigger rapid changes in intracellular calcium levels, apoplastic reactive oxygen species production, and apoplastic pH (Monshausen et al., 2009). These responses can be monitored using specific fluorescent reporters imaged by confocal microscopy (Martinière et al., 2018; Li et al., 2019a; Nietzel et al., 2019). This method has notably been used in the shoot apical meristem where it was shown that the response of the auxin transporter PIN1 to mechanical forces relies on a transient Ca^2+^ response that could be monitored using the fluorescent reporters R-GECO1 and GCaMP6f (Li et al., 2019a). These sensors are thus very promising tools to study the rapid responses of cells to mechanical perturbations. However, it remains to be shown if they can also be used to assess internal stress levels, notably during growth, which happens on slower timescales.

### The rise of single-cell approaches to study mechanics

These last couple of years have seen the rapid development of single cell approaches, including live-cell imaging methods. In developmental mechanobiology, single-cell systems represent a simpler model to study the role of mechanical forces in cellulo, avoiding the additional complexity brought about by the tissue context (e.g. chemical signals, impact of neighboring cells, complex mechanical stress patterns). Recent studies have used such single-cell approaches to assess the relative contributions of both cell geometry and cortex tension to cortical microtubule behavior (Colin et al., 2020; Durand-Smet et al., 2020). In these studies, wall-less plant cells, also called protoplasts, were confined in microfabricated wells of various shapes and sizes (Figure 4A). The protoplasts were either placed under hyperosmotic conditions (i.e. with a reduced cortex tension) or under hypoosmotic conditions (i.e. with an increased cortex tension), and cortical microtubule orientation was then analyzed (Figure 4, B and C). In cells confined in rectangular microwells and exhibiting a reduced cortex tension, cortical microtubules tended to align with the long axis of the cell (Colin et al., 2020; Durand-Smet et al., 2020). In contrast, using microwells of a similar shape, in cells with an increased cortex tension, cortical microtubules mainly aligned with the shortest axis of the cell, which also corresponds to the principal stress direction in these cells (Colin et al., 2020).

One of the main features of protoplasts is their capacity to regenerate a whole organism from a single cell. To investigate this process, microfluidic-based systems were recently designed and adapted to follow protoplast development. In these systems, protoplasts are trapped in chambers, where they are immobilized. Contrary to the previous system, nutrient medium can circulate between chambers, allowing long-term kinetic experiments to be performed. Using such a system combined with a microscope set-up, a recent study investigated the influence of photoperiod on the growth of the moss *Physcomitrium patens* (Sakai et al., 2019). By adding hormones to the circulating medium, the authors also observed the induction of leafy buds (Figure 4D; Sakai et al., 2019). In another study, a microfluidic platform was designed with microelectrodes, coupled with electrical impedance spectroscopy, to study primary cell wall regeneration at the single-cell level (Chen et al., 2020; Figure 4E). In this study, cells displaying a completely regenerated cell wall exhibited higher impedance values (i.e. dielectric properties) compared to nascent protoplasts (Chen et al., 2020). This system also allows researchers to discriminate between several cell wall mutants and wild-type cells, thus providing a new tool for phenotypic analyses (Chen et al., 2020).

There are a number of limitations to such approaches. For example, the cell wall of a protoplast regenerates but may have different properties compared to that of cells in a multicellular context. In addition, it is not clear that properties deduced from experiments on individual isolated cells can be easily applied to cells in their native tissues and organs. Thus, data obtained from single-cell experiments should be backed-up by in vivo analyses, when possible. Furthermore, slight differences in the experimental design may influence the physico-chemical environment of the cells and ultimately have strong impacts on the conclusion. However, this also represent on opportunity. Indeed, if unexpected differences are found, researchers can take advantages of minimal systems, since there are fully controlled, to understand which variables differentially affected the results. Altogether, these single-cell approaches, combined with live-cell imaging and microfluidic methods, open new opportunities to test biological hypothesis in a highly controlled manner.

## Key challenges for live plant cell imaging and possible solutions

### Inherent difficulties in imaging plant cells

#### Apoplast

The plant apoplast (i.e. space outside the plasma membrane) can be described as a “microscopist’s nightmare”, as it represents one of the most formidable challenges for plant cell biologists in term of imaging. Indeed, plant cells are embedded in a thick cell wall often made of highly autofluorescent and impermeable materials. The presence of thick cell walls limits observations of the plasma membrane and the cell cortex in TIRFM. Nonetheless, TIRFM/VA-TIRF has been successfully used to study plant tissues with relatively thin walls, such as the root elongation zone, root hairs, hypocotyls, and young leaves (Konopka et al., 2008; Konopka and Bednarek, 2008; Gronnier et al., 2017; Johnson and Vert, 2017; Platre et al., 2019; Johnson et al., 2020, 2021; Narasimhan et al., 2020; Smokvarska et al., 2020; Bayle et al., 2021). Plant cell biologists also have to face a sometimes impermeable apoplast, which drastically limits the possibility to exogenously add fluorescent compounds. This is one reason why the field is heavily dominated by the use of genetically encoded constructs and fluorescent proteins. The apoplast is also a highly acidic environment, which strongly affects most fluorescent proteins. The solution is to use pH resistant fluorescent proteins, but established sensors that work in the cytoplasm, such as hormone or calcium sensors, may have to be re-engineered to work in such an environment. The apoplast can be very rich in proteases, which often destabilize proteins. A possible solution would be to remove cryptic protease target sites in synthetic reporters. However, in many cases, such sites are not precisely known, which largely preclude such strategies at the moment.

#### Autofluorescence

Plant cells and tissues are very rich in pigments and are highly autofluorescent. In addition, the autofluorescence due to phenolic and carotenoid compounds, as well as chlorophyll and chromophores, spans a wide range of wavelengths. This high autofluorescence can mask true signals, often decreases the signal-to-noise ratio, and complicates automated image analyses. One solution is to use tissues with minimal autofluorescence. For example, root tip cells do not have chlorophyll and also have less autofluorescence in their cell walls because they are undifferentiated. Alternatively, it is possible to use spectral unmixing or fluorescence lifetime imaging to separate true signals from autofluorescence.

#### Cytoplasmic streaming

Plant cells have very active cytoplasmic streaming, which means that intracellular trafficking is fast and difficult to follow using fluorescence microscopy. To circumvent this problem, it is possible to image undifferentiated cells, which have weaker cytoplasmic streaming than highly differentiated cells. Another solution, which was recently introduced to study endocytosis, is to reduce the dynamics of the system by rapidly lowering its temperature (Wang et al., 2020; Johnson et al., 2021). Finally, one can also use fast imaging systems such as spinning disk confocal microscopy, TIRFM, or light sheet microscopy (Table 1).

### A quest to image plant cells and tissues in their native conditions

#### Gravity

Plants grow according to the gravity vector, with positive gravitropism for the root and negative gravitropism for the shoot. This makes it difficult to image certain parts of the plant over a longer period of time, for example when imaging the root tip. In most microscope set-ups, the slides are mounted horizontally, which blocks the gravitropic response. The use of vertical stage microscopes, which allow roots to grow along the gravity vectors, facilitates the dynamic analyses of cell division, as well as studying the gravitropic response and cell elongation (von Wangenheim et al., 2017; Fendrych et al., 2018; Marhava et al., 2019; Serre et al., 2021). Alternatively, light sheet microscopy also allows roots to grow vertically while performing live imaging (von Wangenheim et al., 2016; Ovečka et al., 2018).

#### Light

Plants need light to develop, and they use signaling pathways/photoreceptors to respond to many wavelengths of light. The use of laser beams to excite fluorescent proteins often also triggers these signaling pathways. This is particularly problematic when studying light responses but may also confound other results. It is thus important to carefully confirm that a given response is not affected by the imaging conditions. Interestingly, yellow fluorescent proteins (YFP) are excited by green light (around 514 nm), a wavelength that plants are mostly blind to. YFP derivatives have thus proven to be highly popular among plant biologists. Another solution is to use highly sensitive microscopy techniques to limit the amount of light treatment and thus the activation of light-sensitive pathways (i.e. spinning disk confocal microscopy, light sheet fluorescence microscopy—see Table 1). Finally, for very long imaging experiments, it may be necessary to add lighting above the microscope stage to mimic the day/night cycle.

#### Soil and air

The root system naturally grows in heterogeneous soil, while the aerial parts of the plant grow in the air. However, plant biologists usually mount their plants in homogeneous medium, most often liquid or agar-based. Transparent soil solutions exist (Ma et al., 2019a), and microfluidic devices are becoming increasingly diverse to mimic particular growth conditions, even when heterogeneous (Stanley et al., 2018; Guichard et al., 2020; Yanagisawa et al., 2021). However, this is clearly an area that needs to be further developed in the future. In particular, imaging samples in the air is difficult, as the samples can dry out and the microscope objectives are often not adapted for this type of imaging.

## Conclusions and future prospects

The examples described above illustrate that live imaging of plant cells is challenging on multiple levels. There is no perfect set-up: Live imaging experiments always involve a series of compromises. For example, it is beneficial to have very bright labeling in order to limit photobleaching and generate images with high contrast. For genetically encoded reporters, bright labeling is often associated with strong expression levels. However, strong expression of such probes can deeply perturb the system under study. Thus, one should strike a delicate balance between expression and sensitivity. There is, of course, room for improvement. New fluorescent reporters should be developed that are less toxic, fully reversible, with better dynamic range, and more quantitative. Very often, the development of such tools is highly empirical. They necessitate significant investments in terms of wet lab experiments and can take years of work, with no guarantee of success. However, when new sensors or reporters become available, they can tremendously benefit their fields of study. The use of molecular simulation and in vitro protein evolution are starting to boost the rational design of sensors. We thus envision that the building of new genetically encoded reporters, as well as the production of new dyes, will significantly enhance our ability to image various aspects of plant cell biology in the future.

The choice of a microscopy technique is also a matter of compromise to match the method with the spatio-temporal scale of the system under study. However, it has become increasingly clear that many biological phenomena happen at multiple scales that impose feedback on each other. It is likely that plant biologists will increasingly need “scale-bringing” technologies able to image biological systems at multiple scales. For example, such systems could combine super-resolution capabilities with a wide field of view to study entire organs, or they may be able to perform ultrafast imaging over long periods of time. With advances in electronics, particularly the development of detectors and cameras that are extremely sensitive, such “scale-bridging” technologies are becoming a reality (Clark et al., 2020). The imaging set-up should also allow plants to grow in an environment that is as native as possible in term of light, growing medium, temperature, orientation, the laser power received for imaging, and so on. Various microfluidic devices tailored to the study of precise plant biology phenomena are already emerging (Grossmann et al., 2018; Clark et al., 2020). Given the relatively low cost and high versatility of microfluidic systems, we expect that they will become more and more common in plant live imaging experiments.

Finally, it is clear that image analyses and quantifications are formidable problems that will require multidisciplinary solutions. Some of these solutions may be widely applicable to many projects, such as computational tools to analyze cytoskeleton properties or cell contours. In contrast, in many cases, relevant image analyses will require dedicated scripts and algorithms to answer specific biological questions. It will thus be imperative to build dedicated platforms to host and index these scripts so they can be (re)used, improved, and modified.

In this era of quantitative biology, image analysis of live imaging experiments is often the limiting factor. It will be imperative to train the next generation of plant cell biologists with this in mind.

## Notes

L.C., S.B., and B.L. wrote the paragraph on imaging mechanical properties and responses to forces, R.M-A. and T.V. wrote the paragraph on hormone sensors, M,-C.C. wrote the paragraph on cytoskeleton imaging, Y.J. wrote the paragraph on imaging membranes, the introduction, and discussion. All authors edited the final manuscript. A.B. and B.L. prepared Figure 1, Y.J. prepared Figure 2, all the Tables, R.M-A. prepared Figure 3, and L.C. prepared Figure 4.

The author responsible for distribution of materials integral to the findings presented in this article in accordance with the policy described in the Instructions for Authors (https://academic.oup.com/plcell) is: Yvon Jaillais (yvon.jaillais@ens-lyon.fr).
